# Pathognomonic Features of Olmesartan-Induced Collagenous Sprue Resulting in Severe Small Bowel Malabsorption

**DOI:** 10.7759/cureus.72571

**Published:** 2024-10-28

**Authors:** McKennah A Goshgarian, Jeffrey Laczek, Michael Lustberg

**Affiliations:** 1 Gastroenterology, Uniformed Services University of the Health Sciences, Bethesda, USA; 2 Gastroenterology, Walter Reed National Military Medical Center, Bethesda, USA; 3 Gastroenterology, Sutter Santa Rosa Regional Hospital, Santa Rosa, USA

**Keywords:** autoimmune enteropathy, collagenous sprue, gluten-free diet response, histological diagnosis, medication-associated enteropathy, olmesartan-induced enteropathy, small bowel malabsorption, subepithelial collagen deposition, th2-mediated inflammation, villous atrophy

## Abstract

Collagenous sprue (CS) is a rare autoimmune gastrointestinal disorder characterized by specific histologic changes in the small intestine. It often presents with more severe symptoms and a worse prognosis compared to celiac disease, including significant malabsorption, weight loss, and nutrient deficiencies. Despite treatment with a gluten-free diet, symptom improvement is limited, with only a small fraction of patients responding positively.

This case report highlights the diagnostic challenges and clinical features of CS in a 74-year-old woman, whose symptoms resolved following cessation of olmesartan. The case emphasizes the importance of recognizing medication-induced forms of the disease and outlines the need for targeted management strategies to improve patient outcomes.

## Introduction

Collagenous sprue (CS) is a rare autoimmune gastrointestinal disorder distinguished by specific histologic changes in the small intestine. Similar to other collagenous inflammatory conditions, such as collagenous colitis and gastritis, medications like non-steroidal anti-inflammatory drugs, angiotensin-converting enzyme inhibitors, angiotensin II receptor blockers (ARBs), selective serotonin reuptake inhibitors, and proton pump inhibitors have been associated [1]. 

The association between olmesartan, an ARB, and the development of a sprue-like enteropathy was first reported in the New England Journal of Medicine in 1970 [2]. Olmesartan-associated enteropathy appears to have a female predominance, particularly affecting those aged 50 to 70 years [3]. This disorder presents with severe symptoms, including debilitating malabsorption, significantly impacting patients' quality of life and morbidity. Specific symptoms typically include severe watery diarrhea, muscle wasting, and significant weight loss. Laboratory tests may reveal mixed anemia and hypoalbuminemia, with serologic tests for celiac disease being negative.

Histologically, olmesartan-associated enteropathy is characterized by a thickened subepithelial collagen band and a lymphocytic infiltrate in the mucosa solely within the duodenal mucosa [4,5]. The underlying pathogenic mechanisms are thought to involve chronic Th2-mediated inflammation, similar to other collagenous diseases such as collagenous colitis [4,5]. Notably, most patients experience rapid symptoms and clinical improvement following the withdrawal of the offending medication [3-5].

Here, we present a case that illustrates the diagnostic challenges and management of CS. A 74-year-old woman presented with debilitating symptoms of severe small bowel malabsorption while on olmesartan. Discontinuation of olmesartan led to prompt symptom resolution, weight regain, and resolution of anemia over several weeks. This case underscores the importance of promptly identifying olmesartan-induced CS to prevent severe small bowel malabsorption and improve patient outcomes.

Despite advances in diagnostic techniques, the pathogenesis of CS remains poorly understood, necessitating further research. However, its direct association with olmesartan use cannot be overlooked. This case report summarizes the previous reports and lays out the pathogenic clinical and histologic features of this condition. The relative scarcity of information in the literature underscores the need for increased awareness and understanding within the medical community. Olmesartan is a widely prescribed antihypertensive and is the main offending agent in CS prevalence [3]. With greater recognition of olmesartan’s intense and debilitating symptoms, patient prosperity can be more readily achieved without the detrimental creation of CS. Continued research and collaboration are essential to bring more awareness of olmesartan’s effect on the gastrointestinal system and its impact on patient quality of life.

## Case presentation

A 74-year-old woman with a history of hypertension managed with amlodipine was transitioned to olmesartan and subsequently presented with a six-month history of debilitating gastrointestinal symptoms. Initially experiencing loose bowel movements up to seven times daily and nocturnal fecal incontinence, she developed severe emesis and an alarming weight loss of over 60 pounds. The patient reported no dysphagia, melena, or hematochezia.

Extensive diagnostic workup ensued, including fecal calprotectin and repeated testing for C. difficile infection returned negative results. TTG IgA, IgA, computed tomography (CT) of the abdomen and pelvis, and stool cultures were all negative. Autoimmune etiologies, such as systemic lupus erythematosus (SLE), were considered but all these diagnostic measures did not elucidate the cause of her symptoms. Liver function tests revealed mildly elevated transaminases, raising concerns for hepatic involvement. 

Colonoscopy with random biopsies and terminal ileum intubation was normal.

Upper endoscopy with duodenal biopsies revealed superficial occasional erosions and significant histologic findings: marked villous atrophy, thickened subepithelial collagen deposition, and heightened lymphocytic infiltration in the lamina propria in the duodenum.

**Figure 1:**
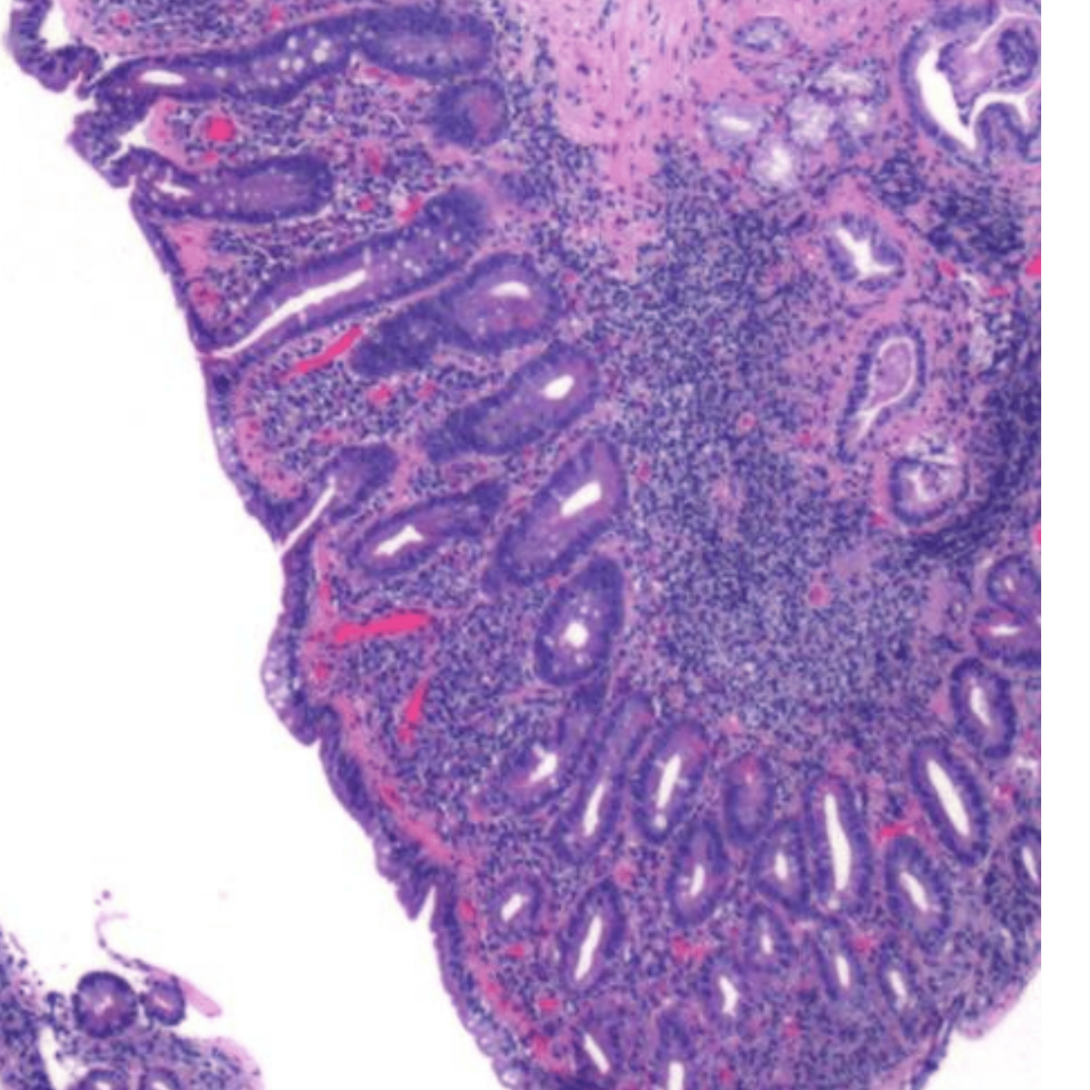
Low-magnification image depicting duodenal mucosa with marked villous atrophy.

**Figure 2:**
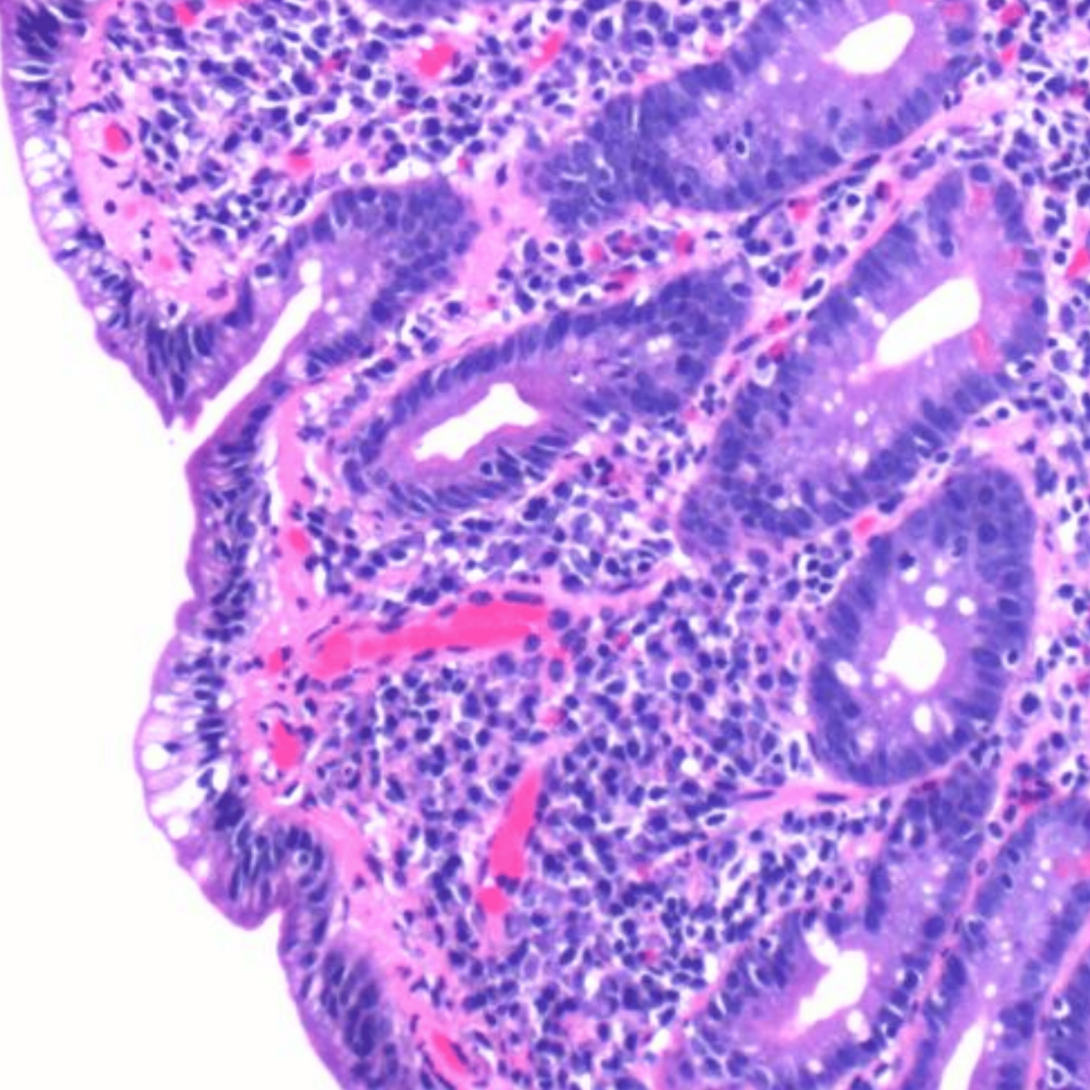
Medium-magnification image depicting duodenal mucosa with marked villous atrophy, thickened sub-epidermal collagen deposition, and increased lymphocytes in the lamina propria and crypts.

**Figure 3:**
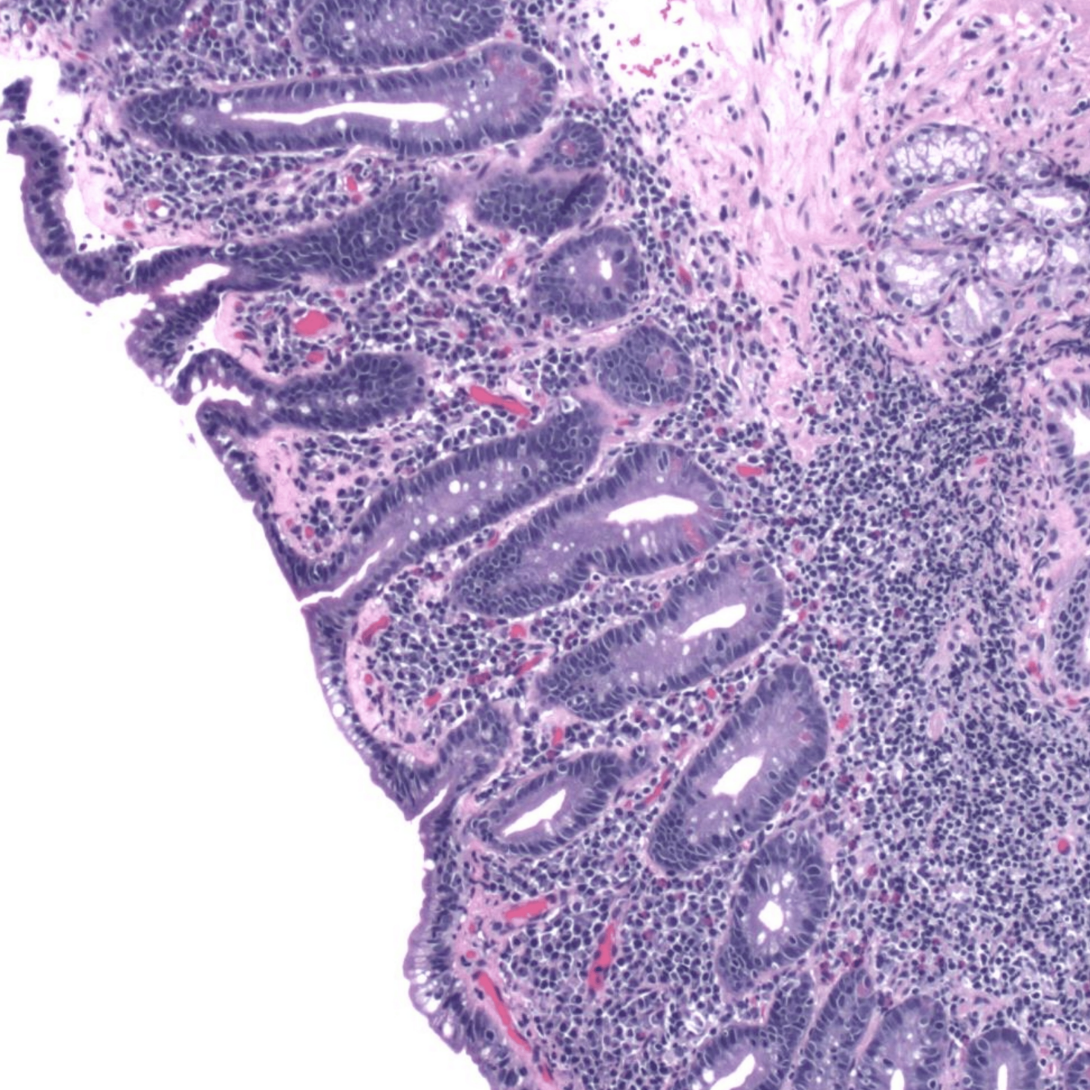
High-magnification image depicting duodenal mucosa with marked villous atrophy, thickened sub-epidermal collagen deposition, and increased lymphocytes in the lamina propria and crypts.

Figures 1-3 depict duodenal mucosa with marked villous atrophy, thickened sub-epidermal collagen deposition and increased lymphocytes in the lamina propria and crypts.

The specific pattern strongly suggested CS as the underlying pathology. Prompt intervention with the cessation of olmesartan resulted in the resolution of symptoms and restoration of severe small bowel malabsorption, as confirmed by subsequent unremarkable colonoscopies and blood tests. The patient's ability to maintain a healthy BMI and remain symptom-free underscores the efficacy of identifying the proven association between olmesartan use, CS and severe small bowel malabsorption. Continued vigilance and multidisciplinary collaboration are essential in optimizing outcomes for patients with these proven gastrointestinal associations and pathologies.

## Discussion

This case highlights the diagnostic complexity and clinical significance of olmesartan-induced CS, particularly in the context of severe small bowel malabsorption. The 74-year-old patient transitioned from amlodipine to olmesartan for hypertension management, subsequently presenting with debilitating gastrointestinal symptoms over a six-month period. Her clinical presentation included severe diarrhea, nocturnal fecal incontinence, and profound weight loss, which are hallmark symptoms of CS.

The extensive diagnostic workup included fecal calprotectin, multiple tests for C. difficile infection, tissue transglutaminase IgA (TTG IgA), total IgA, abdominal and pelvic CT scans, and stool cultures. Despite the comprehensive nature of these investigations, the etiology of her symptoms remained elusive. Autoimmune disorders, such as SLE, were considered but ultimately ruled out.

The breakthrough in diagnosis came through upper endoscopy and repeated colonoscopies with duodenal biopsies. These revealed marked villous atrophy, thickened subepithelial collagen deposition, and increased lymphocytic infiltration in the lamina propria [3]. Such findings are pathognomonic for CS [3].

Prompt cessation of olmesartan led to a remarkable and rapid resolution of the patient's symptoms. This clinical improvement, including the restoration of a healthy BMI and normalization of laboratory parameters, underscores the importance of recognizing medication-induced particularly olmesartan causes of CS, leading to severe bowel malabsorption. The subsequent unremarkable colonoscopies and lab work further confirmed the resolution of severe small bowel malabsorption.

This case emphasizes several critical points for clinical practice as follows:

Medication review

Clinicians should consider a detailed medication history in patients presenting with unexplained gastrointestinal symptoms. Particular attention should be placed on patients concurrently on olmesartan due to its high prevalence of CS leading to small bowel malabsorption [1].

Histological confirmation

Duodenal biopsies are essential for the accurate diagnosis of CS. The characteristic histological features provide definitive evidence needed to distinguish this condition from other gastrointestinal disorders.

Multidisciplinary approach 

Collaboration across specialties, including gastroenterology, pathology, and primary care, is vital for the timely diagnosis and management of complex cases.

Awareness and vigilance

Increased awareness of drug-induced enteropathies can lead to prompt identification and treatment, preventing severe complications such as profound malabsorption and significant weight loss. 

Patient outcomes 

Early recognition and intervention in cases of olmesartan-induced CS can significantly improve patient outcomes, as evidenced by the resolution of symptoms and normalization of nutritional status in this patient.

In conclusion, this case underscores the need for continued vigilance and a high index of suspicion for medication-induced gastrointestinal disorders. Given the widespread use of olmesartan and similar medications, healthcare providers must remain aware of its potential to cause CS resulting in adverse effects such as severe small bowel malabsorption. Quick recognition and discontinuation will optimize patient care and outcomes. Further research and clinical awareness of the pathogenesis and management of CS and its association with medications like olmesartan.

## Conclusions

CS, particularly when drug-induced, presents a rare but significant clinical entity that demands high diagnostic suspicion, especially in patients presenting with severe malabsorption and negative celiac serologies. This case illustrates the importance of thorough histopathological evaluation and medication review in identifying olmesartan as the underlying cause of the patient’s debilitating symptoms.

The prompt cessation of olmesartan not only resulted in symptom resolution but also restored the patient’s nutritional health, highlighting the reversible nature of medication-induced CS. Clinicians must maintain a high index of suspicion for drug-induced enteropathies, particularly in patients on ARBs like olmesartan. Further research is needed to elucidate the full pathogenic mechanisms of this condition and to optimize management strategies, ensuring improved outcomes for future patients.

## References

[1] Hamdeh S, Micic D, Hanauer S (2021). Review article: drug-induced small bowel injury. Aliment Pharmacol Ther.

[2] Weinstein WM, Saunders DR, Tytgat GN, Rubin CE (1970). Collagenous sprue--an unrecognized type of malabsorption. N Engl J Med.

[3] Schiepatti A, Minerba P, Puricelli M, Maimaris S, Arpa G, Biagi F, Sanders DS (2024). Systematic review: clinical phenotypes, histopathological features and prognosis of enteropathy due to angiotensin II receptor blockers. Aliment Pharmacol Ther.

[4] Kamal A, Fain C, Park A, Wang P, Gonzalez-Velez E, Leffler DA, Hutfless SM (2019). Angiotensin II receptor blockers and gastrointestinal adverse events of resembling sprue-like enteropathy: a systematic review. Gastroenterol Rep (Oxf).

[5] Burbure N, Lebwohl B, Arguelles-Grande C, Green PH, Bhagat G, Lagana S (2016). Olmesartan-associated sprue-like enteropathy: a systematic review with emphasis on histopathology. Hum Pathol.

